# Whether Interleaving or Blocking Is More Effective for Long-Term Learning Depends on One’s Learning Strategy

**DOI:** 10.3390/bs15050662

**Published:** 2025-05-12

**Authors:** Jeri L. Little, Jexy A. Nepangue

**Affiliations:** 1 Department of Psychology, California State University, East Bay, 25800 Carlos Bee Blvd, Hayward, CA 94542, USA; jnepangu@ucsc.edu; 2 Department of Psychology, University of California, 1156 High Street, Santa Cruz, CA 95064, USA

**Keywords:** category learning, rule learning, blocking, interleaving

## Abstract

Grouping information into categories enables us to learn, integrate, and apply new information. Presenting items from different categories sequentially (i.e., interleaving) is often more effective than presenting items from a single category sequentially (i.e., blocking), particularly when evaluating learning using memory-based tests. However, blocking can be more effective than interleaving for rule-based learning. Research has investigated optimal sequence (interleaving vs. blocking) for category learning when participants can either memorize or find rules, finding an interaction between learning strategy and sequence; that is, when memorizing, interleaving is more effective than blocking for classifying based on similarity, but when trying to find a rule, blocking is more effective than interleaving for classifying based on the rule. The goal of the present experiments was to replicate and extend this finding by examining transfer immediately after learning and then at a delay of about 48 h. The present results replicate the interaction between sequence and strategy, and show that the pattern persists over a delay. The results also suggest that some rule-based learning may be more resistant than memory-based learning to forgetting. These findings have educational implications for structuring learning as a function of strategy or learning goals.

## 1. Introduction

Grouping information into categories enables us to learn, integrate, and apply new information. Students taking statistics classes need to learn when certain formulas and procedures are necessary. Students in geology courses learn to categorize rocks so that they can later classify specific examples they have not seen. Students often learn categories from examples, and when learning categories of information from examples, effective learning may depend on how those examples are sequenced. Much previous research has shown that presenting items from different categories sequentially (i.e., interleaving) is better for learning than presenting items from the same category sequentially (i.e., blocking; see reviews by Brunmair & Richter, 2019; Firth et al., 2021). However, this advantage of interleaving over blocking depends on the features of the materials and the task. For example, when there is a clear rule and/or items have low within-category similarity, blocking can outperform interleaving (e.g., Noh et al., 2016; Sorensen & Woltz, 2016). Additionally, participants can approach a single task with different strategies, and the optimal sequence can depend on the strategy a learner uses in a task (e.g., memorization vs. rule finding)—at least on tests provided immediately after training (Little et al., 2025). The goal of the present study is to examine optimal sequence (interleaving vs. blocking) as a function of instructed strategy (to memorize or to find a rule) in long-term learning. 

### 1.1. Optimal Sequence for Learning Depends on Strategy

Interleaving items from different categories is often better for learning than blocking those items by category (see reviews by Brunmair & Richter, 2019; Firth et al., 2021). This benefit has been shown in a variety of domains, including the learning of artists’ styles (Kornell & Bjork, 2008), naturalistic stimuli (Wahlheim et al., 2011), math (Rohrer & Taylor, 2007), and simple motor skills (Wulf & Shea, 2002). In the case of visual information, the advantage of interleaving over blocking is often attributed to interleaving’s affordance for discrimination—the discriminative contrast hypothesis (Birnbaum et al., 2013; Kang & Pashler, 2012). That is, when items in different categories have between-category similarity, seeing items from different categories in sequence allows a learner to pick out the critical features that differentiate categories (see also Carvalho & Goldstone, 2015, sequential attention hypothesis).

However, sometimes blocking is more effective than interleaving. For example, when items are defined by a rule that one needs to abstract but within-category similarity is low and/or between-category similarity is high, blocking items from that category is often advantageous because it allows learners to find the critical feature (or combination of features) that items in a category have in common (e.g., Carvalho & Goldstone, 2014b, 2015, 2017; Kurtz & Hovland, 1956). For example, Carvalho and Goldstone (2014b) used “blob” stimuli made up of curvilinear segments; a single curvilinear segment defined the category membership. They manipulated the similarity of items across categories to be high (i.e., blobs had largely the same shape within and between categories) or low (i.e., there were a variety of shapes within and between each category). They showed an interaction between sequence and similarity for performance on novel transfer items. Specifically, there was an advantage of interleaving over blocking for transfer classification when similarity was high, but an advantage of blocking over interleaving when similarity was low. In addition to interacting with the level of between- and within-category similarity, sequence has been shown to interact with other variables to influence learning. For example, Noh et al. (2016) found that sequence interacts with category structure; with a rule-based category structure, blocking outperformed interleaving, but with an information-integration-based category structure (i.e., that would rely more on exemplar-based than rule-based processes), interleaving outperformed blocking.

Related to the research by Noh et al. (2016), researchers have also found sequence to interact with the strategy that a learner uses to learn in a single task (Goldwater et al., 2018; Little et al., 2025). Specifically, in a series of experiments, Little et al. (2025) had participants study letter strings from four different categories in either a blocked or an interleaved order. The letter strings were individualized, but their category designation followed a rule pertaining to the last letter (e.g., items that ended with “t” were plants; those that ended with “k” were tools; those that ended with “b” were clothing; those that ended with “p” were animals). In the blocked condition, participants saw all of the items from a given category sequentially (e.g., plant—spaksvot, plant—coteset, plant—quitinoct). In the interleaved condition, participants saw items from a given category intermixed with items from the other categories (e.g., plant—spaksvot, animal—hazamap, tool—blasimark). On a transfer task, participants had to classify items that looked similar to the trained items but for which the last letter would designate a different category on the basis of the rule (e.g., spaksvok). The idea, based on the research by Little and McDaniel (2015a), was that memorizers would classify the items based on similarity (e.g., spaksvok is a plant), but rule finders would classify the items based on the rule (e.g., spaksvok is a tool). Indeed, Little et al. found that participants differed in how they classified the items. Critically, when participants self-reported as memorizers, interleaving was more effective than blocking for later classification based on similarity, but when participants self-reported as rule finders, blocking was more effective than interleaving for later classification based on the rule. In Little et al.’s Experiment 2, they instructed participants to memorize or find a rule, and they observed the same pattern (participants were only given strategy instructions before training, not again before the test). That is, for those instructed to memorize during training, later similarity-based classification was facilitated by interleaving compared to blocking, but for those instructed to find a rule during training, later rule-based classification was facilitated by blocking compared to interleaving.

### 1.2. Category Learning over a Delay

Some research has examined the efficacy of interleaving versus blocking for long-term learning, examining performance on tests at delays of at least 24 h (Carvalho & Goldstone, 2014a, 2017; Dobson, 2011; Eglington & Kang, 2017; Linderholm et al., 2016; Rohrer et al., 2015; Zulkiply, 2013; Zulkiply & Burt, 2013). The general finding is that interleaving tends to outperform blocking, and while performance tends to be worse (overall) after a delay, the magnitudes of the effects shown immediately and at a delay tend to be comparable (i.e., no interaction between sequence and delay, see Firth et al., 2021). For example, Rohrer et al. (2015) examined interleaved versus blocked practice for math problems (graphs and slopes) and examined performance at one day and 30 days. They found an advantage of interleaving over blocking, and that effect was not significantly different at a 30-day delay. Similarly, Zulkiply (2013) examined interleaved versus blocked practice for texts presented aurally. They found an advantage of interleaving over blocking, and that effect was not significantly different at the one-week delay. Eglington and Kang (2017) examined interleaved versus blocked practice for categories of chemicals with a test delayed by two days (no immediate test). They found that interleaved practice outperformed blocked practice on a test at a two-day delay. 

As mentioned in the earlier section, interleaving does not always outperform blocking on immediate tests, and this has implications for performance on delayed tests. Carvalho and Goldstone (2014a) examined both immediate and delayed transfer using the previously mentioned “blob” stimuli. Their Experiment 1a showed that although high-similarity items benefitted from interleaving over blocking for transfer performance on an immediate test, low-similarity items did not (the blocking advantage shown by Carvalho & Goldstone, 2014b, was not replicated). Nevertheless, the pattern of results was largely consistent for transfer items on the delayed test (24 h), again failing to reveal an interaction between sequence and delay (but see Linderholm et al., 2016, who did show evidence of an interaction between sequence and delay when combined with a testing manipulation).

Thus, most of the time, although performance is lower on a delayed test than on an immediate test, the relative effects of interleaving versus blocking shown on delayed tests are comparable to the effects shown on immediate tests. However, to our knowledge, none of the studies investigating category learning at a delay have used a rule-based category structure (and a task necessitating abstraction of that rule) that resulted in blocking outperforming interleaving on an immediate test. Thus, it is critical to know whether superior learning under blocked conditions would persist over time.

One can gain some insight into the retention of rule-based learning from the problem-solving literature. Abstracting a rule is like solving a problem. Evidence suggests that if a learner solves a problem, their memory for the solution is, to some extent, protected from forgetting (Dominowski & Buyer, 2000). Specifically, Dominowski and Buyer (2000) compared those who solved problems to those who were given the solution. A week later, the “solvers” remembered the solution better than the participants who were given the solution (although see Weisberg & Alba, 1981, who showed forgetting over time with the nine-dot problem). First, Dominowski and Buyer’s results suggest that rule-based performance may not be worse at a delay (which may be different from memory-based category learning). Second, if performance is not worse at a delay, it is also unlikely that the advantage of blocking over interleaving would change from an immediate to a delayed test, but this assumption warrants testing.

In summary, although performance in category learning tasks is often lower on a delayed test than on an immediate test, the relative effects of interleaving versus blocking shown on delayed tests tend to be comparable to those shown on immediate tests. However, to our knowledge, none of the studies investigating category learning at a delay have used a rule-based category structure and a task necessitating abstraction of that rule that resulted in blocking outperforming interleaving. Thus, it is critical to know whether superior learning under blocked conditions would persist over time.

### 1.3. The Present Research

The present research examines the interaction effect between sequence and instructed strategy on performance immediately and after a delay of about 48 h. The present Experiment 1 intends to replicate Little et al.’s (2025) Experiment 2 with a different sample and experimental context (mix of student and non-student participants, online vs. student participants, in-person). The present Experiment 2 extends the research by examining whether the effects persist over a delay, and how performance changes after re-training.

In the present experiments, participants studied letter strings from four different categories (from Little et al., 2025, experiments) in either an interleaved or blocked sequence. Prior to training, participants were either instructed to memorize or to find a rule. At the end of the first session, participants classified transfer items (without any further strategy instructions). In the present Experiment 2, during a second session about 48 h later, participants again classified the transfer items. Participants then completed a re-training phase. Finally, they classified those same transfer items for a third time.

We were most interested in performance on transfer items. First, following from research by Little et al. (2025), we expected that those told to memorize would tend to classify transfer items based on similarity to trained items, and those instructed to find a rule would tend to classify transfer items based on the rule. Second, for those instructed to memorize, interleaving would be more effective than blocking for classifying those transfer items based on similarity; for those instructed to find a rule, blocking would be more effective than interleaving for classifying based upon the rule. Finally, for those instructed to memorize, we expected the benefit of interleaving over blocking to persist when tested later, consistent with prior literature (Firth et al., 2021), but the predictions for rule-abstraction over time are less clear. Borrowing from the problem-solving literature, the rule—once found—may not be easily forgotten (Dominowski & Buyer, 2000). Another idea that would predict little forgetting is that figuring out a solution leads to deep encoding (e.g., Jacoby, 1978). In either case, we would expect to see little forgetting over time, and no interaction between sequence and delay for rule finders. However, although the general rule (i.e., last-letter-designated category membership) may be protected from forgetting, we wagered that specifics of the rule (e.g., “t” means plant, “b” means clothing) could be forgotten or mixed up, resulting in apparent forgetting on a delayed test. Thus, we added a re-training phase, followed by a third transfer test. If forgetting occurred, the responses during retraining would provide insight into the speed of relearning (i.e., given a refresher; c.f. Carvalho & Goldstone, 2014a). We expected that the advantage of blocking over interleaving would remain after re-training.

## 2. Experiment 1

### 2.1. Method

#### 2.1.1. Participants and Design

We recruited 201 participants (96 females, 97 males, 2 non-binary; average age = 32.5, *SD* = 11.84, range = 17 to 64) from psychology courses at California State University, East Bay (*n* = 62) and MTurk (www.mturk.com, *n* = 139). Students received 1 point towards their Research Participation Requirement at California State University, East Bay, for every 50 min (or any part thereof) they participated in. MTurk participants received USD 3 for their participation.

Little et al.’s (2025) Experiment 2, which most closely resembles the present experiment, revealed a large interaction effect (n_p_^2^ = 0.10), with an effect size for those instructed to memorize of *d* = 0.65 and for those instructed to find a rule of *d* = 0.64. In order to have 80% power to observe an effect size of *d* = 0.65 (alpha = 0.05), we needed 39 participants per group (156 total; G*Power 3.1, Faul et al., 2007). We collected data from extra participants to allow for cases we would need to remove. To maintain data integrity, we included attention checks, a training test to assess whether participants were trying to learn, and multiple open-ended questions to help distinguish real participants from bots and to help identify duplicate responses.

This experiment utilized a 2 × 2 between-subjects design. The independent variables were instructed strategy (find rule vs. memorize) and learning sequence (interleaved vs. blocked). Performance for transfer items was assessed based on participants’ classification according to the instructed strategy during the training phase (i.e., memorization strategies were assessed for similarity-based transfer; rule-abstraction strategies were assessed for rule-based transfer).

#### 2.1.2. Materials

Sixteen letterstrings previously used by Little et al. (2025) were used in the present experiment. There were four categories, with four letter strings in each category. The last letter of each letter string determined its category. Plants ended in “t” (i.e., quitinoct, ollinat, spaksvot, coteset). Tools ended in “k” (i.e., lontsink, yallinuk, martwok, blasimark). Clothing ended in “b” (i.e., jollinub, rigelwib, thadtelb, zabarab). Animals ended in “p” (e.g., grasavep, hazamap, flavanop, peetikip).

Transfer items were created to look identical to trained items, except that the last letter was switched with that from another category. Specifically, “t” and “k” were always switched, and “p” and “b” were always switched to minimize the difference in how the words sounded if pronounced. For example, for the trained item “plant—quitinoct”, the transfer item would be “quitinock”. The idea is that the participants who received the rule instructions during training would classify words based on the last letter (e.g., quitinock would be identified as a tool). Those who received the memorization instructions would classify based on the beginning of a word (e.g., quitinock would be identified as a plant).

#### 2.1.3. Procedure

Participants completed this experiment on their own devices. They were instructed to learn words from four categories: plants, animals, tools, and clothing. They were told that they would see each word six times to help facilitate their learning, and that attention check trials would occasionally occur to check their engagement. They were also told not to write anything down. Participants were randomly assigned to a strategy condition (find rule or memorize). Participants in the find rule condition were given the following instructions pertaining to their strategy:

“Rule-based strategies involve trying to come up with a reason for why items are in each category (e.g., trying to find a pattern in letters, sounds, etc. that shows up in all of the words in the category, breaking the code).”

Participants in the memorization condition were told the following pertaining to the strategy they should use:

“Memorization strategies might involve repeating an item over and over to yourself or coming up with a trick for remembering an item (e.g., associating something you already know with those new words, making up a rhyme to remember all of the items in a category, coming up with acronyms using the first letters of the items in the category, etc.).”

Participants were asked to re-state the instructions and then provided with a summary of the instructions. After the instruction manipulation, participants were randomly assigned to the interleaved or blocked sequence condition. In the interleaved sequence, the 16 items from the four different categories were presented in a mixed format (e.g., plant—spaksvot, animal—hazamap, tool—blasimark). Participants saw the 16 items in six blocks, with the items presented in a random order for each block. Starting in the third block, participants were given attention check trials (four in each block, one for each item). For the attention check trials, participants had to provide the category of an item presented the item before the last by choosing among the four categories. After choosing their answer, they were told the correct category designation. In the blocked condition, training consisted of six blocks of the four items from a given category, then six blocks of the four items from another category, and so forth, until all four categories were presented. Attention checks began in the third block for each category. In both conditions, items were shown for 6 s each time they were presented. Attention checks were self-paced.

After the training session, participants completed the training test in which they were instructed to classify the items they had learned by selecting one of the four trained categories. The test was self-paced, and no feedback was provided. Next, participants completed the transfer test in which they were instructed to classify new items. They were not told whether they should classify the items based on similarity or the rule, but our assumption based on previous research was that they would tend to classify based on the instructions they were given for training (Little et al., 2025). The test was self-paced, and no feedback was given. After participants completed the transfer test, they were told that they could have implemented a variety of strategies in this task. Participants were then asked to self-report their strategy on a scale of 1–7, where 1 = *solely memorization* and 7 = *solely trying to find a rule.* Lastly, participants were asked to describe a reason for the grouping of words if any were found.

At the end of the session, participants’ demographic information was collected. They were asked if they had taken part in a study with similar materials and if yes, to explain. They were also asked about distractions, computer problems, and whether they wrote any information down. Most importantly, participants were reassured that their answers would not affect their compensation.

### 2.2. Results and Discussion

The data for the experiments in this paper, including the reasons for participant removal, can be found at Open Science Framework (OSF) (https://osf.io/z8b69/?view_only=71fa1b74f58a490a84ccc6b73f8a82d1, data posted 28 March 2025). 

We removed 34 participants (12 CSUEB students, 22 MTurkers) from the analysis because they reported writing things down, reported having done the task before, their answer to the instruction summary and/or strategy question appeared to be copied and pasted from the internet or did not answer the question, or it appeared that they had done the study more than once (due to similar responses and/or IP addresses). This left 167 participants, with 38 in the rule–interleaved condition, 45 in the rule–blocked condition, 41 in the memorize–interleaved condition, and 43 in the memorize–blocked condition.

#### 2.2.1. Manipulation Check

At the end of the task, we asked participants to rate their strategy on a 1–7 Likert scale where 1 = *solely memorization* and 7 = *solely trying to find a rule*. To assess the instruction manipulation’s effectiveness, we conducted an independent-samples *t*-test on self-reported strategies as a function of instructed strategy. We found that those told to abstract the rule provided numerically—but not significantly—higher ratings (*M* = 4.6, *SD* = 2.3) than those told to memorize (*M* = 4.1, *SD* = 2.1), *t*(165) = 1.51, *p* = 0.133. Another way to assess whether the instruction manipulation succeeded was to examine the relationship between instructions and the classification of transfer items. Controlling for sequence condition, instructions (coded 1 for memorize, 2 for find rule) predicted rule-based categorization, *r*(164) = 0.24, *p* = 0.002, and similarity-based categorization, *r*(164) = −0.24, *p* = 0.002.

#### 2.2.2. Performance for Trained Items

We conducted a 2 (strategy: find rule vs. memorize) × 2 (sequence: interleaved vs. blocked) ANOVA on performance for trained items. Participants performed numerically—but not significantly—better on the trained items if they had received the interleaved schedule (*M* = 0.85, *SE* = 0.03) than the blocked schedule (*M* = 0.79, *SE* = 0.02), *F*(1, 163) = 2.40, *p* = 0.124, η_p_^2^ = 0.01. There was no main effect of instructed strategy (find rule, *M* = 0.83, *SE* = 0.02; memorize, *M* = 0.81, *SE* = 0.02), *F*(1, 163) = 0.74, *p* = 0.390, η_p_^2^ = 0.01, and there was no interaction, *F*(1, 163) = 0.78, *p* = 0.380, η_p_^2^ = 0.01.

#### 2.2.3. Performance for Transfer Items

**Figure 1:**
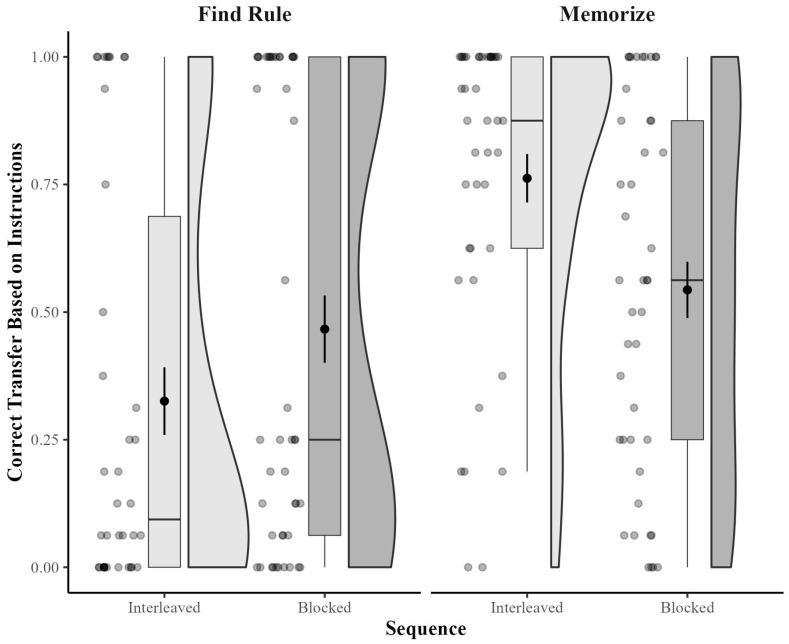
Correct categorization of transfer items as a function of instructed strategy and sequence in Experiment 1. Box plots display the interquartile range (IQR), with the whiskers extending to the minimum and maximum values within 1.5 times the IQR. The black circles within the box plots represent the mean for each cell, with the error bars representing +/−1 SEM. Violin plots depict the density of individual participants’ accuracy scores, with wider sections indicating higher density. Individual participant scores are provided as a scatterplot to the left of the box plots.

The most critical measurement for the present paper was performance on transfer items. Critically, correct performance on transfer items reflected participants’ instructions for training. For those instructed to find a rule during training, correct classification was based on adherence to the rule. For those instructed to memorize during training, the correct classification of transfer items was based on similarity to the trained item (i.e., same letter stem). As demonstrated in Figure 1, there appears to be an interaction between strategy instructions and sequence such that for rule finders, blocking is more effective than interleaving, but for memorizers, interleaving is more effective than blocking.

We analyzed these transfer data using a 2 (strategy: find rule vs. memorize) × 2 (sequence: interleaved vs. blocked) ANOVA. Sequence (interleaved vs. blocked) did not significantly affect correct classification, *F*(1, 163) = 0.43, *p* = 0.515, η_p_^2^ = 0.003 [interleaved *M* = 0.54, *SE* = 0.04; blocked *M* = 0.51, *SE* = 0.04]. However, those instructed to memorize did significantly better at correctly classifying the transfer items based on similarity (*M* = 0.65, *SE* = 0.04) than did those instructed to find a rule classify items based on the rule (*M* = 0.40, *SE* = 0.04), *F*(1, 163) = 18.68, *p* < 0.001, η_p_^2^ = 0.10.

Importantly, we found a significant interaction between strategy and sequence, *F*(1, 163) = 9.16, *p* = 0.003, η_p_^2^ = 0.05. Using independent-samples *t* tests, we conducted planned paired comparisons to assess the effectiveness of interleaving versus blocking for those instructed to memorize and those instructed to find a rule. For those instructed to memorize, interleaving was significantly more effective (*M* = 0.76, *SD* = 0.30) than blocking (*M* = 0.54, *SD* = 0.36) for classifying based on similarity, *t*(82) = 2.99, *p* = 0.002, *d* = 0.65. For those instructed to find a rule, blocking was numerically—albeit not significantly—more effective (*M* = 0.47, *SD* = 0.44) than interleaving (*M* = 0.33, *SD* = 0.41) for classifying based on the rule, *t*(81) = 1.50, *p* = 0.138, *d* = 0.33).^1^

It should be noted that across conditions, answers that were correct based on the rule accounted for 31% of the responses, and answers that were correct based on similarity accounted for 56% of responses. Answers that were errors (i.e., the two remaining possible responses) accounted for only 13% of the responses (chance would be 50%), suggesting that even when participants did not follow instructions, they seemed to classify based on either memory or the rule the majority of the time, which might reflect individual differences in predisposition to memorize or find rules that superseded the instructions, a point we will return to in the General Discussion. 

Although MTurk participants generally outperformed the students, *F*(1, 167) = 5.98, *p* = 0.016, η_p_^2^ = 0.04, participant type did not interact with sequence (*p* = 0.515), strategy (*p* = 0.324), or the interaction of sequence and strategy (*p* = 0.869) to influence performance on transfer items.

#### 2.2.4. Discussion

The results of Experiment 1 mostly replicated those reported by Little et al. (2025). Most critically, there was an interaction between sequence and strategy such that for those instructed to memorize, interleaving was more effective than blocking, but for those instructed to find a rule, blocking was numerically more effective than interleaving, although in the present study, it was a non-significant small-to-medium effect (*d* = 0.33). The finding that interleaving was more effective than blocking for classification based on similarity is consistent with previous research (e.g., Kornell & Bjork, 2008). The finding that blocking is numerically more effective than interleaving for classification based on an articulable rule is also consistent with past research (e.g., Noh et al., 2016). However, the finding that both patterns could be present within the same materials—simply as a function of instructions—is more novel.

The goal of Experiment 2 was to examine whether this interaction would replicate and persist over a delay with a larger sample that would reveal a significant benefit of blocking over interleaving for those instructed to find a rule. To our knowledge, this would be the first study to examine the persistence of rule-based classification over time.

Consistent with prior literature (Firth et al., 2021), we would expect that for those instructed to memorize, interleaving would be more effective than blocking, and this effect would be comparable on a delayed test. However, the predictions for those instructed to find a rule were less clear because no research has investigated the persistence of rule-based classification over time, at least not in a task in which the participant needed to find the rule. We hypothesized that the rule may not be easily forgotten (c.f., problem-solving, Dominowski & Buyer, 2000), and even if the specifics of the rule were (e.g., which letter indicated each category), relearning might be faster for participants who had initially had blocked instruction than for those who had initially had interleaved instruction. For this reason, in Experiment 2, we included a transfer test at the beginning of the second session and then again after a re-training session.

## 3. Experiment 2

### 3.1. Method

#### 3.1.1. Participants

Participants were recruited from MTurk (www.mturk.com). In the first session, 400 individuals participated (148 female, 249 male, 1 non-binary, 2 failed to report), aged 20–67 (*M* = 36.4, *SD* = 9.9). They were paid USD 3 for their participation in the first session.

The 319 participants who classified more than 50% of the trained items correctly at the end of Session 1 (regardless of other problems with their responses) were recruited to return two days later (81 were not asked to return). We recruited participants based on training performance because we believed participants learning fewer than 50% were likely bots or participants who were mindlessly guessing, and we initially believed that this would be a sufficient quality check given that we needed to deploy the task for the second session within 24 h of the end of data collection for the first session. We later removed more participants based on open-ended responses and other criteria, as discussed in the results section. Participants were incentivized to return for the second session with a notification when the study’s second session was ready (in a note attached to a USD 0.50 bonus), and the 293 participants who returned were paid an additional USD 3 for their participation.

From the whole sample of 400 participants (including those who were not invited to return or who were invited but did not return), we removed 108 from the analyses because they reported writing things down, reported having done the task before, their answer to the strategy questions and/or question about instructions appeared to be copied and pasted from the internet or did not answer the question, or it appeared that they had done the study more than once (due to similar responses and/or IP addresses). This left 61 participants in the rule–interleaved condition (*n* = 50 who returned for Session 2), 72 in the rule–blocked condition (*n* = 57 who returned), 79 in the memorize–interleaved condition (*n* = 63 who returned), and 80 in the memorize–blocked condition (*n* = 58 who returned). 

**Power considerations.** We aimed to reveal reliable differences in sequence for both those instructed to find a rule and those instructed to memorize. Little et al.’s (2025) Experiment 2 showed an effect size for those instructed to find a rule of *d* = 0.64 and for those instructed to memorize of *d* = 0.65, but Experiment 1 in the present paper revealed effect sizes for those instructed to find a rule of *d* = 0.33 and for those instructed to memorize of *d* = 0.65. The lower average effect size was shown for rule finders (*d* = 0.49). To accommodate a smaller effect size, we modified our power calculations. Thus, to achieve 80% power (alpha = 0.05, two-tailed) to detect an effect of *d* = 0.49, we aimed for 67 participants per group. Unfortunately, we underestimated how many participants would need to be removed, so we ended up with less than 80% power for rule-finders for Session 2. Nevertheless, post-hoc power analyses revealed that given the sample that remained for Session 1, we had 80% power to detect an effect of *d* = 0.49 (alpha = 0.05, two-tailed) in the rule condition. Given the sample that remained for Session 2, we had 71% power to detect an effect size of *d* = 0.49 (alpha = 0.05, two-tailed) in the rule condition. We had 97% power to detect an effect of *d* = 0.64 in the memorize condition for Session 1 and 94% power to detect that effect for Session 2.

#### 3.1.2. Design

This experiment utilized a 2 (strategy: find rule vs. memorize) × 2 (sequence: interleaved vs. blocked) × 3 (time: Session 1 vs. Session 2—beginning vs. Session 2—end) mixed-subjects design. As in Experiment 1, we manipulated instructed strategy (find rule vs. memorize) and learning sequence (interleaved vs. blocked) between subjects. Additionally, we assessed transfer performance at three points (within subjects): the end of Session 1, the beginning of Session 2 (about 48 h later), and the end of Session 2 (after another round of training). As in Experiment 1, participants were only given instructions for training, with the expectation that those instructions would influence how they classified items during the three transfer tests. Thus, as in Experiment 1, performance was assessed based on the classification of transfer items according to the instructed strategy (i.e., rule-finding strategies were assessed based on rule-based transfer; memorization strategies were assessed based on similarity-based transfer).

#### 3.1.3. Materials and Procedure

**Session 1.** The materials were the same as those used in Experiment 1. Participants completed this experiment on their own devices. The procedure for Session 1 was the same as that used in Experiment 1 (training, test for training items, test for transfer items), except that participants did not rate their strategies at the end of Session 1 because we did not want to alert them to the other strategy. Instead, participants were given an open-ended question, which differed between the two instruction conditions. Participants in the rule condition were asked if they figured out the rule. Participants in the memorization condition were asked to explain what they did to memorize the items—or, if they did something different—to explain that strategy. We collected 99% of the responses within 24 h of deploying Session 1.

**Session 2.** Participants who answered more than 50% of training items correctly at the end of Session 1 were recruited to participate in Session 2. Session 2 was deployed 48 h after we deployed Session 1. Within an hour of deployment, we sent out a bonus of USD 0.50 with a message indicating that the second session of the study was ready to complete, and data collection occurred for up to 48 h. Ninety-eight percent of participants returned within 72 h. The average delay between the start of the first session and the start of the second session was 49 h (range 24 h to 85 h).

In the second session, participants were first tested on the 16 transfer items (self-paced, no feedback). Then, they completed re-training trials (four cycles, all in an interleaved sequence). Instead of presentation trials (i.e., used in Session 1), the re-training trials were test–feedback trials akin to the attention checks used in Session 1. Participants were presented with the trained items and asked to choose the correct category. After choosing the answer, the correct category was provided. 

After the re-training phase, participants completed the third transfer test with all 16 transfer items. After the transfer test, they provided a rating pertaining to their strategy (1 = *memorization*, 7 = *find a rule*). Specifically, they were asked, “On a scale of 1 to 7, where ‘1’ means that you were relying solely upon memorization of the items and ‘7’ means that you were solely trying to find a rule, give a rating for what you were doing when you were learning the items on the first day of this experiment?”

At the end of the session, participants’ demographic information was collected. Then, they were asked if they had taken part in a study with similar materials and, if yes, to explain. As this was an online study, they were also asked about any distractions and computer problems they experienced while partaking in the study as well as whether they wrote anything down. Most importantly, they were reassured that their answers would not affect their compensation.

### 3.2. Results

#### 3.2.1. Manipulation Check

At the end of Session 2, we asked participants to rate their strategy on a 1–7 Likert scale. Indeed, participants who were instructed to find a rule provided ratings that were more rule-like (*M* = 5.0, *SD* = 2.1) than did participants instructed to memorize (*M* = 3.6, *SD* = 2.3), *t*(225) = 4.71, *p* < 0.001, *d* = 0.62. Similar to our findings in Experiment 1, instructions also predicted how participants classified transfer items. Controlling for sequence condition, we found that instructed strategy (coded memorize = 1, find rule = 2) was associated with rule-based classification, *r*(289) = 0.31, *p* < 0.001, and similarity-based classification, *r*(289) = −0.26, *p* < 0.001, on the transfer task at the end of Session 1.

#### 3.2.2. Session 1 Performance

Performance for trained items. We conducted a 2 (strategy: find rule vs. memorize) × 2 (sequence: interleaved vs. blocked) ANOVA on performance for trained items. Participants performed better on the training items if they had received the interleaved schedule (*M* = 0.87, *SE* = 0.02) compared to the blocked schedule (*M* = 0.81, *SE* = 0.02), *F*(1, 288) = 4.90, *p* = 0.028, η_p_^2^ = 0.02. There was no main effect of instructed strategy (memorize, *M* = 0.82, *SE* = 0.02; find rule, *M* = 0.85, *SE* = 0.02), *F*(1, 288) = 1.33, *p* = 0.249, η_p_^2^ = 0.005, and there was no interaction, *F*(1, 288) = 0.91, *p* = 0.342, η_p_^2^ = 0.003.

**Figure 2:**
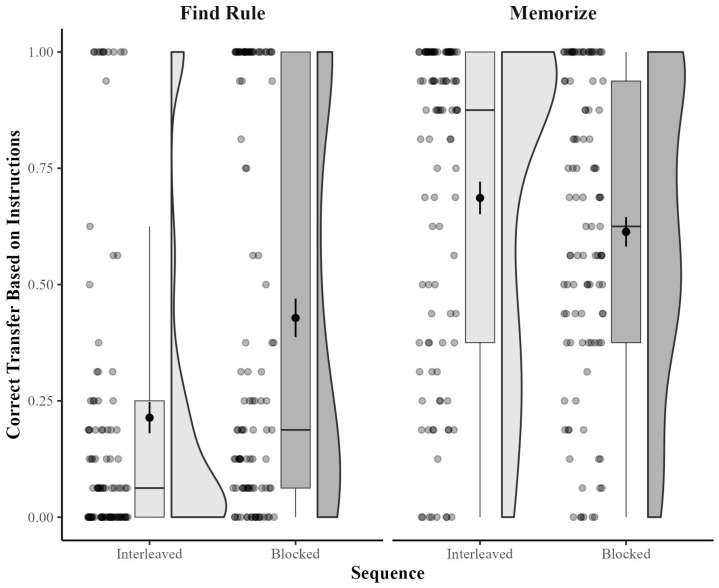
Correct categorization of transfer items as a function of instructed strategy and sequence at the end of Session 1 in Experiment 2. Box plots display the interquartile range (IQR), with the whiskers extending to the minimum and maximum values within 1.5 times the IQR. The black circles within the box plots represent the mean for each cell, with the error bars representing +/− 1 SEM. Violin plots depict the density of individual participants’ accuracy scores, with wider sections indicating higher density. Individual participant scores are provided as a scatterplot to the left of the box plots.

Performance for transfer items. Critically, we wanted to examine the potential interaction between strategy and sequence at the end of Session 1 (i.e., to replicate Experiment 1). As shown in Figure 2, we found an interaction such that when instructed to find a rule, participants tended to classify correctly based on the rule more often when they had received a blocked sequence than when they had received an interleaved sequence. However, when instructed to memorize, participants tended to classify correctly based on similarity more often when they had received an interleaved sequence than when they had received a blocked sequence. It also appeared that participants instructed to memorize were better at their task (i.e., classifying based on similarity) than rule-finders (i.e., classifying based on the rule).

To test these apparent results, we then conducted a 2 (strategy: find rule vs. memorize) × 2 (sequence: interleaved vs. blocked) ANOVA to assess the effects of strategy and sequence on correct transfer at the end of Session 1 based on the instructions. Indeed, we found a significant main effect of strategy such that participants instructed to memorize classified correctly based on similarity more (*M* = 0.69, *SE* = 0.03) than participants instructed to find a rule classified correctly based on the rule (*M* = 0.42, *SE* = 0.03), *F*(1, 288) = 38.89, *p* < 0.001, η_p_^2^ = 0.12. There was also a main effect of sequence, such that participants classified items correctly more often when they had been blocked (*M* = 0.61, *SE* = 0.03) than when they had been interleaved (*M* = 0.50, *SE* = 0.03), *F*(1, 289) = 6.44, *p* = 0.012, η_p_^2^ = 0.02.

Critically, we also found the predicted interaction between strategy and sequence, *F*(1, 288) = 20.39, *p* < 0.001, η_p_^2^ = 0.07. Specifically, when instructed to find a rule, participants classified items correctly based on the rule significantly more often in the blocked condition (*M* = 0.57, *SD* = 0.43) than in the interleaved condition (*M* = 0.26, *SD* = 0.37), *t*(130) = 4.40, *p* < 0.001, *d* = 0.76. When instructed to memorize, participants classified items correctly based on similarity numerically—but not significantly—more often in the interleaved condition (*M* = 0.73. *SD* = 0.35) than in the blocked condition (*M* = 0.64, *SD =* 0.32), *t*(157) = 1.61, *p* = 0.109, *d* = 0.26.^2^

#### 3.2.3. Performance Across Delay

The analysis of performance across delay uses only those participants who returned for Session 2 and were not excluded for data integrity reasons (*n* = 228).

**Figure 3:**
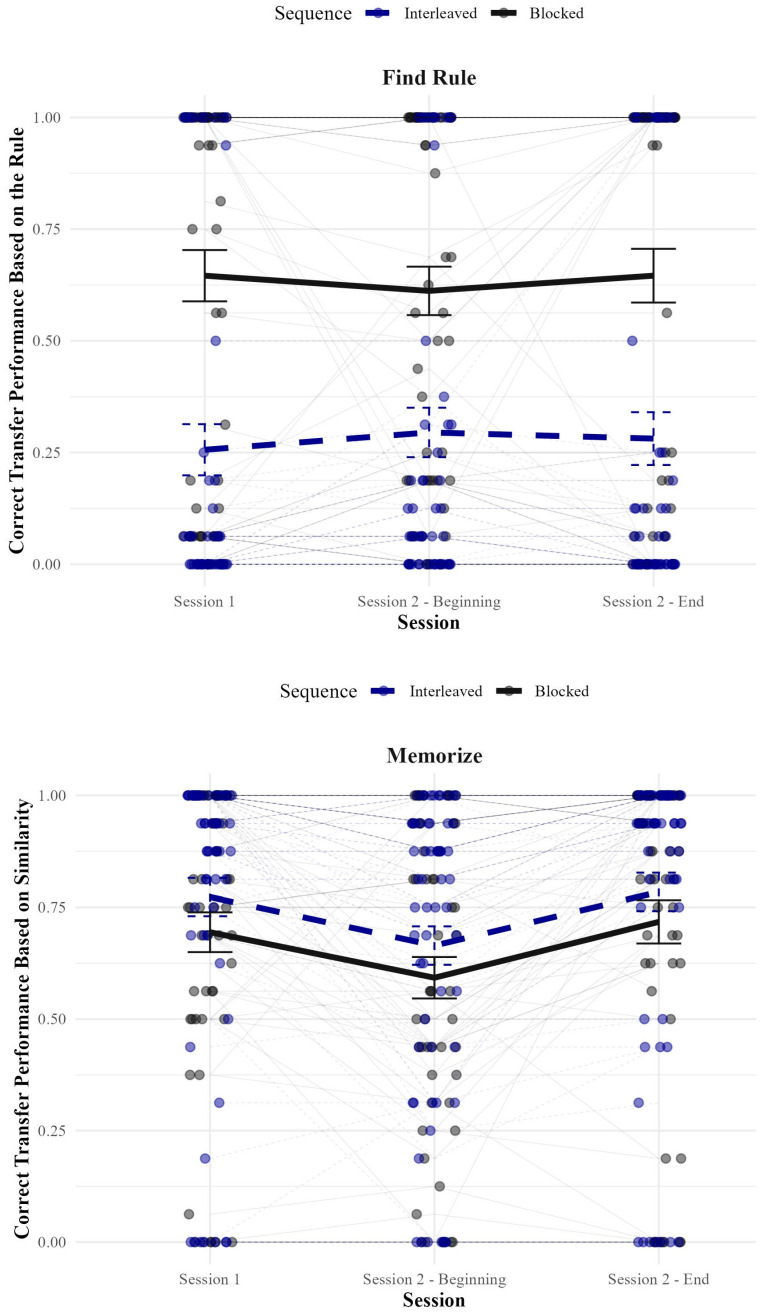
Correct classification based on instructions as a function of strategy instructions and sequence at the end of Session 1, beginning of Session 2, and end of Session 2. The top panel shows transfer performance for those instructed to find a rule. The bottom panel shows transfer performance for those instructed to memorize. Individual participant scores are provided as scatterplots, and performance for a given individual across time is marked with a line. Error bars represent =/− 1SEM. 95% confidence intervals.

Figure 3 shows correct transfer performance based on instructions for those instructed to find a rule (top panel) and memorize (bottom panel) as a function of sequence at all three time points (Session 1, Session 2—beginning, Session 2—end). Figure 3 shows that performance for those instructed to memorize—but not for those instructed to find a rule—dropped from Session 1 to the beginning of Session 2, but rebounded by the end of Session 2. With the exception of the apparent timing effects, it appears that the interaction shown in Session 1 persisted at the beginning of Session 2 and at the end of Session 2.

To assess these apparent effects, we conducted a 2 (strategy: find rule vs. memorize) × 2 (sequence: interleaved vs. blocked) × 3 (time: Session 1, Session 2—beginning, Session 2—end) ANOVA on transfer performance. First, we will describe the main effects. There was a main effect of strategy such that those instructed to memorize classified items based on similarity did better (*M* = 0.71, *SE* = 0.03) than did those instructed to find a rule classify items based on the rule (*M* = 0.45, *SE* = 0.04), *F*(1, 224) = 20.21, *p* < 0.001, η_p_^2^ = 0.08. There was a main effect of sequence such that blocking was better (*M* = 0.65, *SE* = 0.03) than interleaving (*M* = 0.51, *SE* = 0.03) for performance, *F*(1, 224) = 8.61, *p* = 0.004, η_p_^2^ = 0.04. Finally, there was a main effect of time, *F*(2, 436) = 11.12, *p* < 0.001, η_p_^2^ = 0.05. Specifically, performance dropped from Session 1 (*M* = 0.59, *SE* = 0.03) to the beginning of Session 2 (*M* = 0.54, *SE* = 0.03), but then rebounded by the end of Session 2 (*M* = 0.61, *SE* = 0.03). Post-hoc LSD tests confirmed that there was a significant difference between Session 1 and the beginning of Session 2 (*p* < 0.001) and between the beginning of Session 2 and the end of Session 2 (*p* < 0.001), but no difference between Session 1 and the end of Session 2 (*p* = 0.284).

Next, we discuss interactions. Although there was the expected two-way interaction between strategy and sequence, *F*(1, 224) = 20.21, *p* < 0.001, η_p_^2^ = 0.08, there was not a three-way interaction between strategy, sequence, and time, *F*(2, 448) = 0.99, *p* = 0.374, η_p_^2^ = 0.004, indicating that the interaction between strategy and sequence persisted over the delay and re-training period. The average test performance across all three tests was examined using paired comparisons for interleaving versus blocking for those instructed to find a rule and those instructed to memorize. Looking at those average scores, for those instructed to find a rule, blocking was significantly more effective (*M* = 0.63, *SD* = 0.41) than interleaving (*M* = 28, *SD* = 0.40), *t*(105) = 4.54, *p* < 0.001, *d* = 0.88. For those instructed to memorize, interleaving was numerically—albeit not significantly—more effective (*M* = 0.75, *SD* = 0.31) than blocking (*M* = 0.67, *SD* = 0.32), *t*(119) = 1.30, *p* = 0.196, *d* = 0.24.

There was also an interaction between time and strategy, *F*(2, 448) = 8.91, *p* < 0.001, η_p_^2^ = 0.04, suggesting that those instructed to memorize experienced a drop in performance between the first test and the second (which rebounded after re-training), but performance for those instructed to find a rule did not change across time. A one-way repeated-measures ANOVA confirmed that those instructed to memorize showed different performance over time, *F*(2, 240) = 22.04, *p* < 0.001; η_p_^2^ = 0.16; LSD tests revealed that performance went down from Session 1 to the beginning of Session 2, *p* < 0.001, and then up from the beginning of Session 2 to the end of Session 2, *p* < 0.001. There was no effect of time on performance for those instructed to find a rule, however—*F*(2, 212) = 0.84, *p* = 0.841; η_p_^2^ = 0.002.^3^ The interaction between time and sequence was not reliable, *F*(2, 448) = 0.80, *p* = 0.451, η_p_^2^ = 0.004.

#### 3.2.4. Considering Both Experiments

Overall, we replicated the basic pattern of results shown by Little et al. (2025). Specifically, in both experiments, we found an interaction between strategy and sequence such that for those instructed to find a rule, blocking tended to be more effective than interleaving, but for those instructed to memorize, interleaving tended to be more effective than blocking. However, the exact pattern of the results differed a bit between the two studies. In the present Experiment 1, we obtained a significant effect for those instructed to memorize, but not for those instructed to find a rule, and in Experiment 2, we saw the reverse (although the effect was present for those instructed to memorize in the non-parametric comparison). We will return to this inconsistency in the General Discussion. Because the training and test procedures were exactly the same for the studies on the first day, we collapsed over both studies to examine performance for trained and transfer items. A 2 (strategy: find rule vs. memorize) × 2 (sequence: interleaved vs. blocked) ANOVA revealed a significant effect of interleaving (*M* = 0.86, *SE* = 0.02) over blocking (*M* = 0.80, *SE* = 0.02) on trained items, *F*(1, 455) = 7.36, *p* = 0.007, η_p_^2^ = 0.02. There was no main effect of strategy, *F*(1, 455) = 2.00, *p* = 0.159, η_p_^2^ = 0.004, nor was there an interaction, *F*(1, 455) = 1.70, *p* = 0.193, η_p_^2^ = 0.004.

The pattern of results for transfer items reveals a significant interaction of strategy and sequence, *F*(1, 455) = 28.53, *p* < 0.001, η_p_^2^ = 0.06. For those instructed to find a rule, blocking was significantly more effective (*M* = 0.53, *SD* = 0.43) than interleaving (*M* = 0.29, *SD* = 0.39), *t*(213) = 4.34, *p* < 0.001, *d* = 0.59, and for those instructed to memorize, interleaving was significantly more effective (*M* = 0.74, *SD* = 0.33) than blocking (*M* = 0.60, *SD* = 0.34), *t*(241) = 3.07, *p* = 0.002, *d* = 0.39.^4^

#### 3.2.5. Discussion

The present experiment examined transfer performance as a function of strategy (find rule vs. memorize), sequence (interleaved vs. blocked), and time (Session 1—end vs. Session 2—beginning vs. Session 2—end). At the end of Session 1, we found an interaction between strategy and sequence such that when participants were instructed to find a rule, they performed better in classifying items based on the rule when they had seen those items blocked than when they had seen them interleaved. However, when they were instructed to memorize, they performed numerically better when they received items in an interleaved versus in a blocked sequence (the difference was significant when assessing with non-parametric tests, which are more robust to the violations of normality in our data). This interaction pattern persisted across time, with performance dropping for those instructed to memorize—but not for those instructed to find a rule—between the end of Session 1 and the beginning of Session 2, and then rebounding by the end of Session 2. Finding that performance did not drop for those instructed to find a rule is consistent with the findings by Dominowski and Buyer (2000), who showed that people remember their solutions to problems, presumably because figuring out the solution to a problem is memorable (and possibly involves a deeper level of processing, Craik & Lockhart, 1972; c.f., Jacoby, 1978). Taken together, these results suggest that (a) the relative advantage of a given sequence on transfer does not change much over a delay, and (b) even when given a chance to re-train, participants persist in the same strategies.

The pattern of results at the end of Session 1 was reasonably consistent with that shown by Little et al. (2025) in their Experiment 2. However, performance—in general—was better in the present study than in their Experiment 2, especially for those participants who were instructed to find a rule. The pattern of results mostly persisted over time, which is consistent with the findings of other long-term retention and transfer research (e.g., Rohrer et al., 2015; Zulkiply, 2013). 

When considering the results from both experiments (on the first day), we found that interleaving was better than blocking for trained items. This is consistent with the basic spacing effect (Cepeda et al., 2006). For transfer items, the optimal sequence depended on the instructed strategy. For those instructed to find a rule, blocking was more effective than interleaving. For those instructed to memorize, interleaving was more effective than blocking. 

## 4. General Discussion

Across two experiments, we found an interaction effect between strategy and sequence on learning. Specifically, for those instructed to find a rule, blocking tended to be more effective than interleaving, but for those instructed to memorize, interleaving tended to be more effective than blocking. The present experiment 1 mostly replicated the results of an earlier study (Little et al., 2025, Exp. 2), and the present Experiment 2 extended the results, showing that the pattern of results persists over a delay and even after a re-training phase.

### 4.1. Optimal Sequence for Learning Depends on Strategy 

The benefit of interleaving over blocking for those instructed to memorize was large in Experiment 1 (*M_difference_* = 0.22, *d* = 0.65) and relatively small in Experiment 2 (*M_difference_* = 0.09, *d* = 0.26). Similarly, for those instructed to find a rule, the benefit of blocking over interleaving was relatively small in Experiment 1 (*M_difference_* = 0.14, *d* = 0.33) but large in Experiment 2 (*M_difference_* = 0.31, *d* = 0.76). There was no difference in the methods at the point at which participants took the test, so to what extent can we trust these results? The differences between interleaving and blocking in the present Experiments 1 and 2 are well within the 95% confidence intervals for differences in means reported by Little et al. (2025) for their Experiment 2, which used the same materials and procedure (for those instructed to memorize, 95% CI_difference_ [0.05, 0.30]; for those instructed to find a rule, 95% CI_difference_ [0.06, 0.36]). What is clear across both of the present studies is that the interaction is reliable, with a large effect. This interaction between strategy and sequence has been demonstrated in the two studies here as well as in Little et al.’s (2025) Experiment 2. Additionally, Little et al. (2025) reported two experiments examining optimal sequence as a function of self-chosen strategy, and both of those studies showed a similar pattern of results (with those findings having implications for the role of individual differences in strategy, a point we return to later).

The pattern of results for those instructed to memorize is mostly consistent with research showing that interleaving tends to be more effective than blocking when tests involve similarity-based classification that relies on memory (e.g., painting styles, Kornell & Bjork, 2008; physics problems, Samani & Pan, 2021). With regard to the performance over a delay, the findings are consistent with past research that has shown that the benefit of interleaving over blocking tends to persist from immediate to delayed tests (Firth et al., 2021). A point worth mentioning is that in the present research, it is not clear whether interleaving—that is, mixing up items from different categories—is what provides a memory advantage. In the present study, the interleaved condition also included spaced repetitions because each item was shown six times (plus once as an attention check). Under these circumstances, the spacing effect might explain enhanced performance for similarity-based classification (Cepeda et al., 2006). Indeed, when collapsing across both studies, we obtained a benefit of interleaving over blocking for trained items as well, evidencing a spacing effect for trained items. That is, had we only presented each item once, it is unclear whether there would have been any advantage of interleaving. Also worth mentioning here is that although the transfer test for those instructed to find a rule was clearly a test of transfer (not just memory), the “transfer” test for those instructed to memorize was more like a basic memory test, being a *very* near transfer test. 

The pattern of results found here for those instructed to find a rule is consistent with research by Noh et al. (2016), who showed that when one needs to abstract an articulable rule, blocking is more effective than interleaving. With regard to the performance over a delay, the findings that rule-based learning is resistant to forgetting and maintains an advantage of blocking over interleaving at a delay are novel. This effect has not been shown in the category-learning literature. These results are consistent, however, with the idea that once a learner has solved a problem, their memory for the solution receives some protection from forgetting (Dominowski & Buyer, 2000). Specifically, Dominowksi and Buyer showed that learners who solved a variety of published problems (e.g., horse trade, farmer, water jars, nine-dot) had almost perfect memory for the solutions one week later. Interestingly, Dominowski and Buyer compared memory for “solvers” to memory for participants who were exposed to the answer, and memory for solvers was far better. Although there is not much research on the retention of category rules over time, Dominowksi and Buyer’s results suggest that it would depend on whether the learner would have to figure out the rule themselves (i.e., solve the problem) or if the rule was given to them (c.f., Jacoby, 1978). In fact, in some research in which items are defined by a rule, interleaving outperforms blocking, but in most cases, these are situations in which the rule is given to participants (e.g., math formulas, Rohrer & Taylor, 2007). It would be interesting to see if such rule-based learning was resistant to forgetting.

### 4.2. A Role for Individual Differences

Previous research has revealed individual differences in propensities to find rules versus memorize them in a variety of cognitive tasks (e.g., category learning, e.g., Craig & Lewandowsky, 2012; causal learning, e.g., Zaman et al., 2023; function learning, McDaniel et al., 2014; multiple-cue judgements, e.g., Juslin et al., 2003; skill acquisition, e.g., Bourne et al., 2010; see Little & McDaniel, 2015b for a review), including category learning studies with materials similar to the ones used here (Goldwater et al., 2018; Gouravajhala et al., 2020; Little & McDaniel, 2015a; Little et al., 2025; Liu et al., 2023; Wahlheim et al., 2016). In fact, Little et al. (2025, Exps. 1a/b) showed that when given general learning instructions (i.e., all participants were instructed “to learn”, without the mention of memorization or rule finding), participants chose their own strategy, with some reporting that they oriented towards memorization and others reporting that they oriented towards rule-abstraction (see also Goldwater et al., 2018; Little & McDaniel, 2015a). Little et al.’s (2025) pattern of results when looking at self-selected strategies mimicked the pattern shown in the present experiments. For memorizers, interleaving tended to outperform blocking, but for rule-abstractors, blocking tended to outperform interleaving. 

In the present research, some learners instructed to memorize found rules, and some learners instructed to find rules memorized. Figure 1, Figure 2 and Figure 3 all show many cases in which participants who were told to find the rule failed to classify any items correctly, indicating that they classified all of the items based on similarity. Similarly, those figures also all show many cases in which participants who were told to memorize failed to classify any items correctly based on similarity, indicating that they classified all of the items correctly based on the rule.

So, to what extent can participants be instructed to use a given strategy? The manipulation check for Experiment 1 failed to reveal a significant effect of instructions on strategy ratings. However, the manipulation check in Experiment 2 was reliable (as were the correlations between instructed strategy and rule-based and similarity-based transfer performance in both Experiments 1 and 2). Nevertheless, the relatively small effects of instruction on ratings suggest that some participants may have used their predisposed strategy in spite of the instructions, perhaps because they believed that their strategy would be more effective for the task. It is also possible that participants tried to use the instructed strategy but changed their strategy (e.g., they were told to memorize but they noticed the rule; were told to find the rule but reverted to memorizing when they could not find it). Of course, it is also possible that participants failed to read the instructions. This last concern was partially mitigated by asking participants to report the instructions before the task and providing feedback (i.e., again instructing them to either memorize or find a rule). It is prudent to consider the role of multiple factors (e.g., task demands, individual propensities, expectations) in how a learner approaches a task (c.f., McDaniel & Butler, 2011).

### 4.3. Additional Limitations

Often, in studies examining long-term retention, participants receive a test only at the long-term retention period (or delay is manipulated between subjects). In order to maximize power, we opted to make delay a within-subjects variable, similar to the procedure used by Carvalho and Goldstone (2014a). Whether the results would be different had participants not taken a transfer test at the end of Session 1 is an open question. It is possible that the transfer test solidified participants’ learning, especially for the rule condition, and had they not had that test, performance would have been much worse in the rule-based condition after a delay (c.f. testing effects, Roediger & Karpicke, 2006). Inconsistent with Carvalho and Goldstone, however, we tested transfer performance twice during the second session: once before re-training and once after. We did this because we wanted to measure the pure retention of learning, but we also wanted to allow for the possibility that learners—particularly those in the rule condition—might remember the general rule but mix up the specific letter–category combinations. Interestingly, we found that they tended to retain the general rule and the specifics, negating our need to analyze re-training performance.

A related question is why we elected to give all participants an interleaved schedule during re-training. First, in the case that performance dropped for rule finders, we wanted to be able to assess how quickly their performance improved. To do so, we needed to use test–feedback trials rather than presentation trials; and when using test–feedback trials, it does not make sense to compare interleaved to blocked re-training because, for interleaved re-training, answers are far less predictable. The second reason is that we wanted re-training to serve as a reminder of past learning, not supplant it. In particular, we did not want people to learn the rule during the second session, which would have been more likely to happen if re-training was blocked. If the reader feels that using interleaving re-training complicates the results, they can consider just the results at the end of Session 1 and at the beginning of Session 2. Considering only the first two transfer tests, the results provide the same interpretation we have put forth so far—Strategy and sequence interact to affect learning, and this pattern does not change over a delay. Additionally, similarity-based performance is lower after a delay, but rule-based performance is not.

Those instructed to find a rule tended to perform worse in classifying based on the rule than did those instructed to memorize perform at classifying based on similarity. Although our goal was not to compare across this variable, this finding suggests that the rule condition entailed a harder task than did the memorize condition. We think that this is because finding the rule—particularly in the interleaved condition—is a harder task than is memorizing, at least in the present context. One might argue that one reason that the memorize task is easier is that learners can correctly classify based on similarity with only partial memory of the trained stimuli (e.g., some of the letters from quitinoct, with the exception of the last letter). Those instructed to find a rule, on the other hand, would need memory of the last letter for each category (and either to have figured out the rule or forgotten all of the other letters); without that knowledge, performance would be poor. Although not necessarily a problem for the current experiments (because our goal was not to compare memorization performance to rule-based performance), it does have some implications for educational contexts, a point we will address in the next section.

Another limitation is that this study only uses one set of materials, and these results may not generalize to other rule-based categories. Specifically, the rule that “last letter determines category membership” is relatively easy to remember, and the specific letters may not have been that much harder to remember. Thus, the “problem-solving” nature of the task is relatively high, but the need for memory to retain the rule is relatively low. We wager that a rule with higher memory demands may not show the same pattern of results, and would likely result in decreased performance over time. Additionally, transfer items that look similar to trained items but that should be classified differently based on a rule may be uncommon in educational contexts (although see Little & McDaniel, 2015b; Little et al., 2025 for examples; and we return to this issue in the next section).

Finally, although we framed this paper as research with application to education, and although we used an educationally realistic delay between learning and test in Experiment 2, the materials used in the present paper lack external validity to educational contexts. Nevertheless, the current results suggest that future research should be conducted with educational materials, and in the next section, we outline possibilities.

### 4.4. Educational Applications

Interestingly, education is often set up to promote blocked learning. For example, textbooks are often ordered in blocks of categorical information, with the idea that you must master one category of information before moving on to the next. But an advantage of interleaving has been shown across a wide array of materials, including painting style (Kornell & Bjork, 2008), naturalistic categories (Wahlheim et al., 2011), math (Rohrer & Taylor, 2007; Samani & Pan, 2021), and even simple motor skills (Wulf & Shea, 2002). Research supporting the advantage of interleaving over blocking over such a wide range of materials would suggest that a blocked sequence is always worse. Our results (and others, e.g., Noh et al., 2016) show that is not the case.

So when might blocking be advantageous? We describe two examples (concepts in psychology, concepts in analogical reasoning). These examples have, to our knowledge, not been empirically tested. As such, we do not know whether the results would align with the ideas we have put forth here, but we think that they would, and we provide them as possible future directions to infer the ecological validity of these results for education. 

In the classroom, there are sometimes situations in which an instructor provides several examples of a concept, with those examples lacking superficial similarity but meant to convey an overarching idea. For example, instructors often teach about encoding specificity (i.e., retrieval success depends on the overlap between cues available at encoding and retrieval, Tulving & Thomson, 1973) in introductory psychology or cognitive psychology courses. In doing so, they tend to provide experiments from a variety of situations, including, for example, Godden and Baddeley’s (1975) research examining context-dependent memory as a function of environment, Eich and Metcalfe’s (1989) research examining context-dependent memory as a function of mood, and Fisher and Craik’s (1977) research examining memory as a function of the match between encoding tasks and transfer tasks. Although students sometimes struggle to learn this concept even when related experiments are presented in a blocked order, we wager that they would fare even worse at abstracting the rule (i.e., what encoding specificity is/how to apply it) if the specific examples were presented far apart and interleaved with other memory concepts (which should promote item-specific memory). Based on the problem-solving literature (Dominowski & Buyer, 2000), it is worth considering that having students try to figure out the connection between these research experiments—although very difficult—might help them to retain the concept (analogous to figuring out the rule in the current research), but this is a question for future research. In the experience of the first author, students often understand the connection between the first two studies (both context effects), but they have a difficult time connecting Fisher and Craik’s study to encoding specificity, instead simply linking it with the idea of levels of processing (i.e., the idea that semantic processing of a stimulus leads to better memory of it than does processing based on sounds or physical characteristics; Craik & Lockhart, 1972), owing to the similarity of the research in those two examples (and one of the authors’ names). 

For this encoding specificity example, a test question analogous to the transfer questions used in the present experiments might look like a levels-of-processing question (e.g., what orienting task should lead to the best performance?) but ask about predictions—based on the encoding specificity principle—for performance on a phonological-type (i.e., sound-based) test. The choices would be orienting tasks that were not explicitly discussed in class. A student who did not fully understand the idea of encoding specificity would likely predict that a semantic orienting task is better, whereas a learner who had understood the concept would choose an orienting task that is more phonological in nature.

The idea that learning often depends on abstracting a (possibly hard to notice) deep structure (or features) while ignoring superficial structure (or features) is a prominent idea in research on expertise (Chi et al., 1981). Experts develop a deep understanding of their topics and answer new problems based on concepts/rules rather than similarity (Chi et al., 1981). In some cases, such rules or concepts may have been instructed, but over time experts likely abstract consistencies and patterns. The juxtaposition between surface similarities and deep similarities is also discussed within the analogical reasoning literature (Gick & Holyoak, 1980, 1983), and the ideas presented here could help make clear predictions for future work with those materials (see also, Richland & McDonough, 2010). 

For example, in a well-known example, Gick and Holyoak provided participants with Duncker’s (1945) “radiation problem”, and they were asked to come up with a solution to destroy a tumor. Prior to the problem, some participants were given a story about a general who wanted to overtake a fortress that shared structural/deep features—but not superficial features—with the tumor problem (i.e., the story introduced a convergence solution for overtaking the fortress that could be used to solve the tumor problem). Gick and Holyoak found that participants struggled in their ability to use the story about the general attacking the fortress to help solve the tumor problem unless they were explicitly given a hint that the two were related. However, in another study, Gick and Holyoak (1983) found that participants were increasingly likely to be able to solve the problem—without a hint—if they had received two stories with the same structural features (i.e., convergence solution) but different superficial features (e.g., one about the general and the fortress and another about a fire chief and a fire). Two superficially dissimilar stories were better than two superficially similar stories. Providing both stories allowed participants to figure out the commonality that actually mattered—that is, the convergence principle. This structural similarity was the “diagnostic feature that did not jump out at them”. Although Gick and Holyoak (1980, 1983) did not examine interleaving versus blocking with their materials, we wager that participants would be more likely to abstract the underlying principle/deep structure if stories with the same rule were blocked rather than interleaved with stories featuring a different principle. One might imagine a transfer question that shared superficial similarities with one study (fortress), but had a problem that necessitated a different (non-convergence) solution that was provided within another set of materials. 

## 5. Conclusions

In conclusion, in the present studies, we showed that the optimal sequence can depend on the strategy that participants use to learn, assessed on tests at both short and long delays. When memorizing, interleaving tends to be more effective than blocking; when trying to find a rule, blocking tends to be more effective than interleaving. This pattern of results persists over a delay, and even after a re-training phase, showing evidence that rule-based knowledge may be more resistant to forgetting than memory-based knowledge. Based on the present results, we suggest that future research explore these ideas with more educationally realistic materials.

## Data Availability

Data are available at https://osf.io/z8b69/?view_only=71fa1b74f58a490a84ccc6b73f8a82d1 (data posted 28 March 2025).

## Notes

1 Following the hypothesis, these were planned comparisons. Because the scores were not normally distributed, we also conducted independent-sample Mann–Whitney U tests to corroborate the interpretation of these planned comparisons. Indeed, for those instructed to memorize, interleaving was better than blocking, *U* = 572.50, *p* = 0.005; for those instructed to find a rule, the difference was unreliable, *U* = 1024.50, *p* = 0.113.

2 We also conducted independent-samples Mann–Whitney U tests to corroborate the interpretation of these planned comparisons. Indeed, for those instructed to find a rule, the difference was reliable, *U* = 3179.50, *p* < 0.001. Additionally, the non-parametic test revealed that for those instructed to memorize, interleaving was better than blocking, *U* = 2581.50, *p* = 0.043.

3 Because we did not see any drop in performance for rule-finders, we did not analyze re-training performance.

4 We also conducted independent-samples Mann–Whitney U tests to corroborate the interpretation of these planned comparisons. Indeed, for those instructed to find a rule, blocking was more effective than interleaving, *U* = 7810.00, *p* < 0.001. For those instructed to memorize, interleaving was more effective than blocking, *U* = 5708.00, *p* = 0.001.
